# Temperature and Recognition Dual Responsive Poly(*N*-Isopropylacrylamide) and Poly(*N*,*N*-Dimethylacrylamide) with Adamantyl Side Group

**DOI:** 10.3390/ma11040473

**Published:** 2018-03-22

**Authors:** Qiujing Dong, Chunhua Luo, Na Li, Jiaxiang Chi, Qingqing Zhang

**Affiliations:** 1 School of Chemistry and Materials Engineering, Fuyang Normal University, Fuyang 236037, China; dongqj1980@163.com (Q.D.); Lina041722@163.com (N.L.); Chijx961103@163.com (J.C.); Zhangqing24958@163.com (Q.Z.); 2 Anhui Provincial Key Laboratory for Degradation and Monitoring of the Pollution of the Environment, Fuyang 236037, China

**Keywords:** temperature responsive, molecular recognition, poly(*N*-isopropylacrylamide), poly(*N*,*N*-dimethylacrylamide), adamntyl groups, β-cyclodextrins

## Abstract

A series of copolymers with an adamantyl side group (poly(NIPAM-co-AdMA) and poly(DMAM-co-AdMA)) were prepared by radical copolymerization of *N*-isopropylacrylamide (NIPAM) and *N*,*N*-dimethylacrylamide (DMAM) with a 2-methyl-2-adamantylmethacrylate (AdMA) monomer. The structure and composition of the as-synthesized copolymers were characterized by Fourier transform infrared (FT-IR) spectroscopy, proton nuclear magnetic resonance (^1^H NMR) spectroscopy, gel permeation chromatography (GPC), thermogravimetric analysis (TGA), and elemental analysis. Temperature and recognition dual responsivity of the copolymers was investigated by cloud point (T_cp_) and dynamic light scattering (DLS), respectively. The results show that the as-synthesized copolymers are a kind of temperature-responsive polymer with a lower critical solution temperature (LCST). T_cp_ was approximately consistent with the critical temperature of intermolecular copolymer association (T_ass_) from DLS. For these copolymers, T_cp_ decreases with increasing content of AdMA unit in the copolymers. After the addition of β-cyclodextrins (β-CD), T_cp_ increases, and the increment of T_cp_ values gradually became large with increasing content of AdMA in the copolymers. It is host-guest molecular recognition of β-CD and adamantyl groups that endows the as-synthesized copolymers with recognition-tunable thermosensitivity.

## 1. Introduction

Temperature-responsive polymers have attracted much attention in recent years due to their broadly envisaged applications, such as separation of biomacromolecules, surface modification, shape memory materials, water management, biomedicine, and sensors [1,2,3,4,5,6,7,8,9,10,11,12]. Temperature-responsive polymers can undergo a coil–globule reversible transition in response to external temperature in a controlled fashion at a certain temperature (lower critical solution temperature, LCST or upper critical solution temperature, UCST) [13]. Among the various classes of temperature-responsive polymers, poly(*N*-isopropylacrylamide) (PNIPAM) is the most widely studied LCST-type polymer and exhibits reversible sharp phase transitions in water at around LCST of 32 °C [14].

It is important and essential to adjust and control the LCST of these polymers for special applications. Random copolymerization with hydrophobic or hydrophilic comonomers is usually used to tune LCST [15,16,17]. Takei reported that the LCST of PNIPAM was increased by the introduction of the relatively hydrophilic acrylamide and lowered by the introduction of the hydrophobic *N*-butylacrylamide [17]. Furthermore, the temperature-responsive properties of a polymer are often affected by solvents, salts, surfactants, pH, polymer end groups, polymer concentration, polymer topology, or the addition of supramolecular hosts [18,19,20,21,22,23,24,25]. Especially, some supramolecular hosts, such as α,β,γ-cyclodextrins (α,β,γ-CD) and cucurbit[8]uril, have been used to modulate thermosensitivity through supramolecular interactions of polymer side groups or end groups with hosts using external triggers or additional compounds [26,27,28,29,30,31]. The Ritter group reported that the thermosensitive properties of NIPAM-based copolymers and acrylamide polymers bearing adamantyl groups could be influenced by β-CD and its derivatives, spacer groups, and concentration [24,32,33,34,35]. The combination of temperature-responsive polymers with supramolecular chemistry, inspired by the molecular mechanisms behind natural systems, is resulting in adaptive and smart materials with unprecedented properties [26,36].

In this article, we synthesized a series of adamantyl-containing poly(NIPAM-co-AdMA) and poly(DMAM-co-AdMA) copolymers. Temperature and recognition dual responsivity of the copolymers was investigated by cloud point (T_cp_) and dynamic light scattering (DLS), respectively. The effect of β-cyclodextrins (β-CD) on the thermosensitivity of the as-synthesized copolymers was investigated and differences in the temperature-responsive properties of poly(NIPAM-co-AdMA) and poly(DMAM-co-AdMA) copolymers were discussed.

## 2. Materials and Methods

### 2.1. Materials

*N*-isopropylacrylamide (NIPAM) was purchased from TCI (Shanghai, China) Development Co., Ltd., and purified by recrystallization from toluene and n-hexane before used. 2-Methyl-2-adamantylmethacrylate (AdMA) was purchased from TCI (Shanghai) Development Co., Ltd. 2,2′-Azodiisobutyronitrile (AIBN) was purified by recrystallization from ethanol. *N*,*N*-dimethylacrylamide (DMAM) and β-cyclodextrins (β-CD) were purchased from Shanghai J&K Scientific Co., Ltd., Shanghai, China. Other materials were received from commercial sources and used directly without further purification.

### 2.2. Preparation of Poly(NIPAM-co-AdMA)

**Scheme 1:**
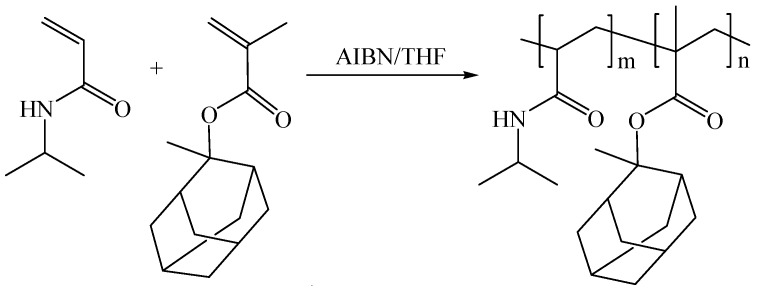
Synthesis of poly(NIPAM-co-AdMA).

A series of poly(NIPAM-co-AdMA) copolymers were prepared by radical copolymerization of NIPAM and AdMA using AIBN as initiator (Scheme 1). Briefly, 2.26 g (20 mmol) of NIPAM, 9.8 mg (0.06 mmol) of AIBN and different feed ratios of AdMA were dissolved in 20 mL of tetrahydrofuran (THF) in 50 mL three-necked bottle. The solution was bubbled with N_2_ for 20 min and stirred at 60 °C for 16 h under N_2_ atmosphere. The solution was poured into 100 mL of diethyl ether and the precipitate was collected by filtering. The obtained crude product was redissolved into THF and precipitated in diethyl ether twice.The purified polymer was dried at 50 °C under vacuum for 24 h to give white powder.

### 2.3. Preparation of Poly(DMAM-co-AdMA)

**Scheme 2:**
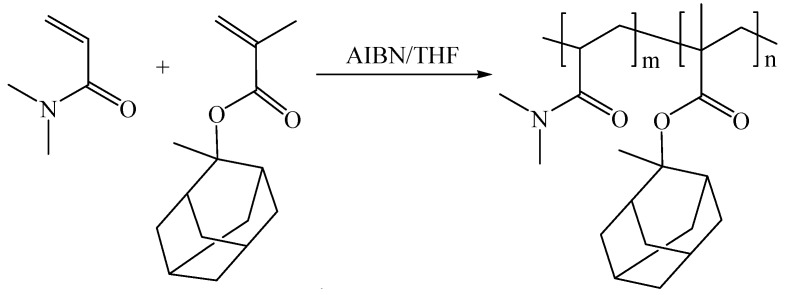
Synthesis of poly(DMAM-co-AdMA).

A series of poly(DMAM-co-AdMA) copolymers were prepared similarly to that of poly(NIPAM-co-AdMA) copolymers using DMAM instead of NIPAM (Scheme 2).

### 2.4. Characterization of Polymers

Fourier transform infrared (FT-IR) spectra were recordedon a 560 FT-IR spectrometer (Nicolet, Waltham, MA, USA) using KBr pellets. Proton nuclear magnetic resonance (^1^H NMR) spectra were measured on a AVANCE AV400 NMR spectrometer (400 MHz, Bruker, Basel, Switzerland) using tetramethylsilane (TMS) as the internal standard at ambient temperature in CDCl_3_. Ultraviolet–visible (UV-vis) spectra were measured on a TU-1901PC spectrophotometer (Beijing Purkinje General, Beijing, China). Gel permeation chromatography (GPC, Waters, Milford, MA, USA) was performed on a Waters Breeze 1525 system equipped with a Waters 2414 detector (35 °C) and a HT4 styragel column (40 °C) using tetrahydrofuran (THF) as an eluent with an elution rate of 1.0 mL/min. The molecular weights were calibrated by a series of polystyrene standards with molecular weights of 1930, 2930, 4910, 10,100, 21,700, 50,000, 123,000, 264,000, 400,000, and 591,000 Da. Thermogravimetric analysis(TGA) was performed on a SDT Q600 Analyzer (TA Instruments, New Castle, DE, USA) at a scan rate of 10 °C/minunder N_2_ atmosphere. The hydrodynamic diameter measurementswere carried out on a 90 plus particle size analyzer (Brookheaven, NY, USA). The elemental analysis (EA) of the polymers was performed on a vario EL cube elemental analyzer (Elementar, Langenselbold, Germany).

### 2.5. Measurement of Temperature Responsive Properties

The temperature responsive properties of the polymers were investigated by both the transmittance at 600 nm and the hydrodynamic diameter of the 5 g/L polymer aqueous solution with respect to the change of the temperature. Transmittance at 600 nm was recorded as a function of the temperature of the polymer solution at the heating rate of 0.5 °C/min on a UV-vis spectrophotometer (Purkinje General 1901) equipped with a PCT-2 Peltier heated pool rack. The hydrodynamic diameter of the polymer was measured by dynamic light scattering with respect to the temperature change. T_cp_ was defined where half of the overall transmittance had changed. T_ass_ was defined where the rapid change of the hydrodynamic diameter had taken place.

## 3. Results and Discussion

### 3.1. Preparation of Poly(NIPAM-co-AdMA) and Poly(DMAM-co-AdMA)

A series of thermosensitive copolymers with an adamantyl side group (poly(NIPAM-co-AdMA) and poly(DMAM-co-AdMA)) were prepared by radical copolymerization of NIPAM andDMAM with the AdMA monomer for 16 hat 60 °C in THF solution using AIBN as initiator (synthesis illustration shown in Scheme 1 and Scheme 2). The polymerization conditions and results are shown in Table 1. For poly(NIPAM-co-AdMA) copolymers, the molar content of adamantyl moiety in the copolymers, determined by elemental analysis according to C and N content in the copolymers, is less than that in the feed, while it was more than that in feed for poly(DMAM-co-AdMA) copolymers. The yield of the copolymers varied from 58 to 74%. The molecular weights of the copolymers were measured by GPC calibrated with polystyrene standards. The number-average molecular weight (Mn) of the as-synthesized copolymers with the adamantyl moiety was between 4000 and 8600 with polydispersity index (PDI, Mw/Mn) varying from 1.7 to 2.5.

### 3.2. Characterization of Poly(NIPAM-co-AdMA) and Poly(DMAM-co-AdMA)

**Figure 1:**
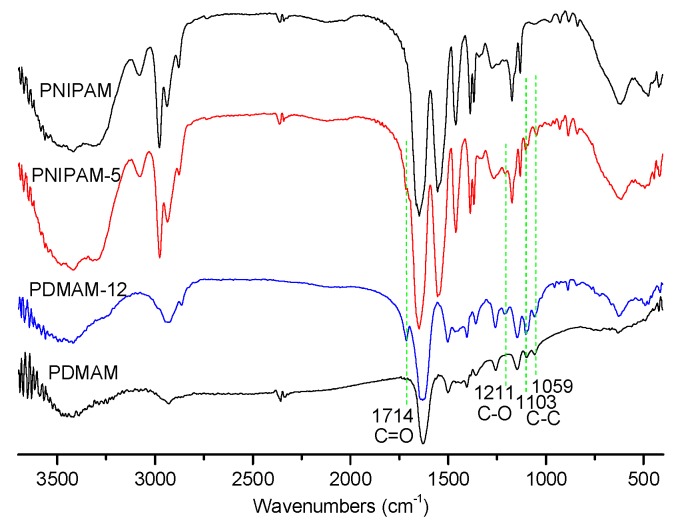
FT-IR spectra of PNIPAM homopolymer, PNIPAM-5 copolymer, PDMAM homopolymer, and PDMAM-12 copolymer.

**Figure 2:**
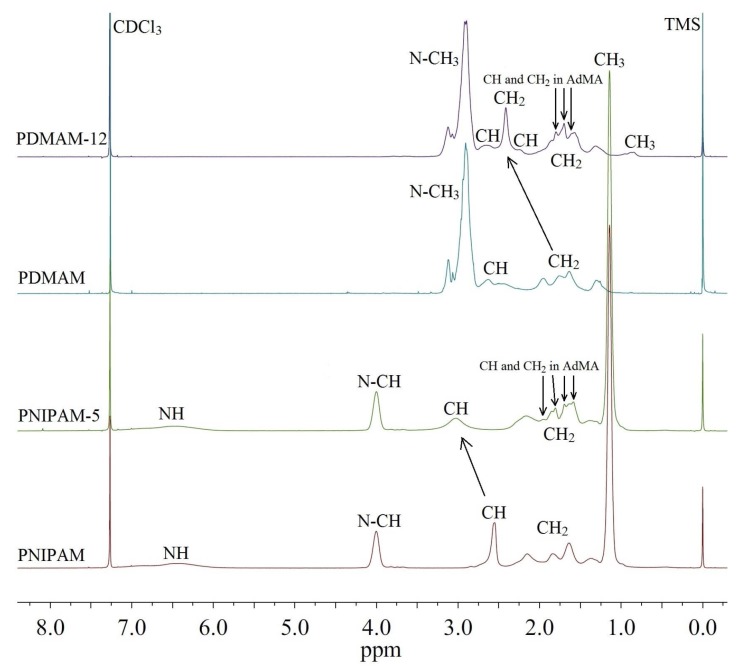
^1^H NMR spectra of PNIPAM homopolymer, PNIPAM-5 copolymer, PDMAM homopolymer, and PDMAM-12 copolymer in CDCl_3_.

The structure of the as-synthesized copolymers (poly(NIPAM-co-AdMA) and poly(DMAM-co-AdMA)) was characterized by FT-IR and ^1^H NMR spectroscopy. Figure 1 shows FT-IR spectra of the PNIPAM homopolymer, PNIPAM-5 copolymer, PDMAM homopolymer, and PDMAM-12 copolymer. It can be seen that the AdMA unit in the copolymers exhibits characteristic absorption bands at 1714 cm^−1^, 1211 cm^−1^, 1103 cm^−1^, and 1059 cm^−1^, which is attributed to the C=O, C–O, and C–C stretching vibrations of the AdMA unit. The characteristic absorption of the AdMA unit in the PNIPAM-5 copolymer is weaker than that in the PDMAM-12 copolymer due to the lower content of AdMA unit in the PNIPAM-5 copolymer compared with the PDMAM-12 copolymer. Furthermore, the ^1^H NMR spectra of PNIPAM homopolymer, PNIPAM-5 copolymer, PDMAM homopolymer, and PDMAM-12 copolymer in CDCl_3_ confirmed their structures (Figure 2). Compared with the spectra of PNIPAM-5 and PNIPAM, the wide and weak resonance signal from 6.20 to 6.80 ppm is assigned to the NH group from NIPAM unit. The resonance band at 4.00 ppm is assigned to the CH group in the isopropyl moiety. The band at 1.25–2.40 ppm is assigned to the –CH_2_– group in the main chain of PNIPAM-5. The resonance peak of the methyl group appears at 1.14 ppm for PNIPAM-5. The resonance peak from the –CH– group in the main chain for PNIPAM-5 shifts downfield to 3.02 ppm due to the introduction of the AdMA unit, which appears in the region of 2.55 ppm for PNIPAM. The protons resonance signals from theAdMA unit are overlapped by –CH_2_– and CH_3_– groups from the NIPAM unit in PNIPAM-5. Similarly, compared with the spectra of PDMAM-12 and PDMAM, the resonance signals of –CH_2_– and –CH– groups from the AdMA unit are overlapped by the –CH_2_– group from the DMAM unit in PDMAM-12. The resonance peak of methyl group from the AdMA unit in PDMAM-12 appears in the region of 0.89 ppm. For PDMAM-12, the partial resonance peak of the –CH_2_– group from the DMAM unit shifts to 2.41 ppm from 1.95 ppm due to the effect of the AdMA unit. So, the peaks at 2.13–2.75 ppm in the spectrum of PDMAM-12 are assigned to –CH– and partial –CH_2_– groups from the DMAM unit, and the other protons resonance signals of –CH_2_– groups from the DMAM unit appear in the region of 1.12–2.10 ppm in which the peaks of–CH_2_– and –CH– groups from the AdMA unit appear. The resonance bands from 2.75 to 3.30 ppm are attributed to the *N*,*N*-dimethyl group from the DMAM unit.

**Figure 3:**
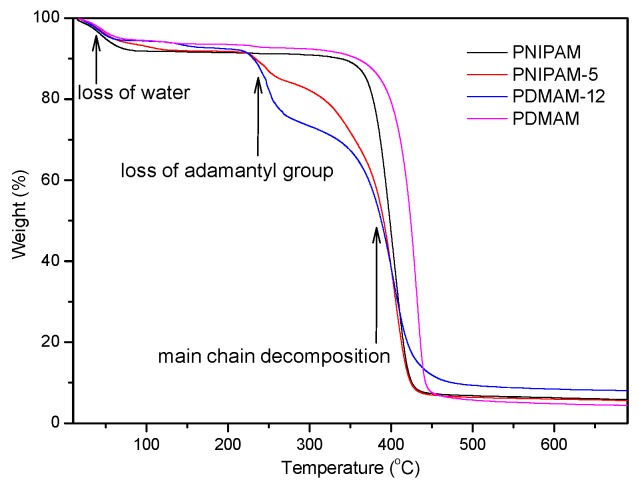
TGA curves of PNIPAM, PNIPAM-5, PDMAM, and PDMAM-12.

Figure 3 shows the thermogravimetricanalysis (TGA) results of PNIPAM, PNIPAM-5, PDMAM, and PDMAM-12. The results indicate that the thermal stability of PNIPAM-5 and PDMAM-12 was decreased due to the introduction of the AdMA unit. The adamantyl side group of the copolymer became lost when the temperature reached 220 °C. The temperature of main chain decomposition was about 350 °C. Compared with PNIPAM-5 and PDMAM-12, PNIPAM-5 showed 10% weight loss of the adamantyl side group at a temperature around 250 °C, which was lower than that of PDMAM-12 with about 20% weight loss.

### 3.3. Temperature and Recognition Dual Responsivity of Poly(NIPAM-co-AdMA) and Poly(DMAM-co-AdMA)

**Figure 4:**
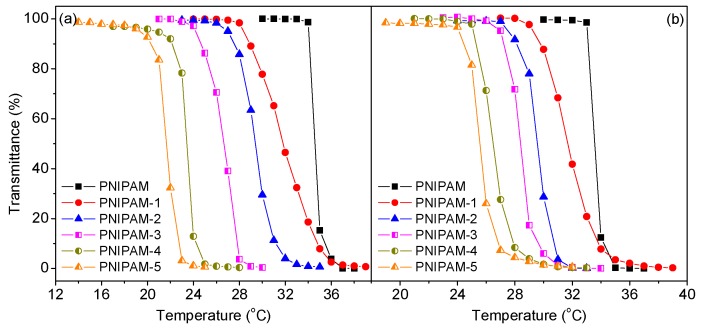
Transmittance as a function of temperature for 5 g/L poly(NIPAM-co-AdMA) aqueous solution without β-CD (**a**) and with β-CD (**b**) observed at a wavelength of 600 nm and a heating rate of 0.5 °C/min.

**Figure 5:**
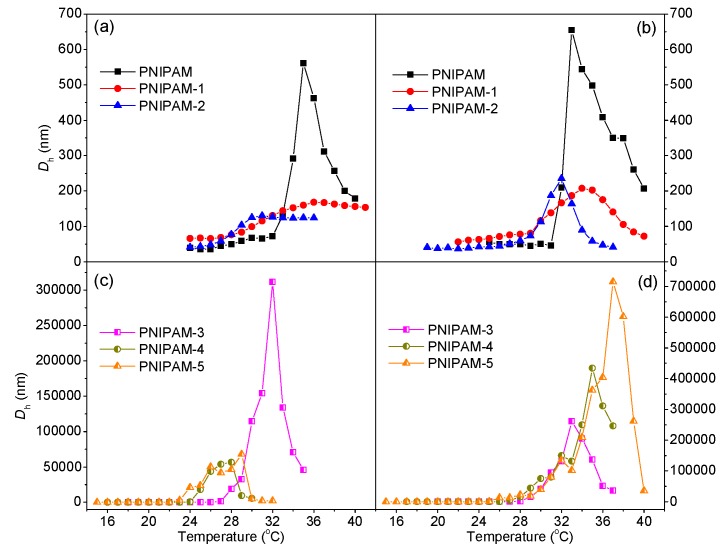
Size distribution of the aggregates of 5 g/L poly(NIPAM-co-AdMA) in water without β-CD (**a**,**c**) and with β-CD (**b**,**d**) as a function of temperature.

**Figure 6:**
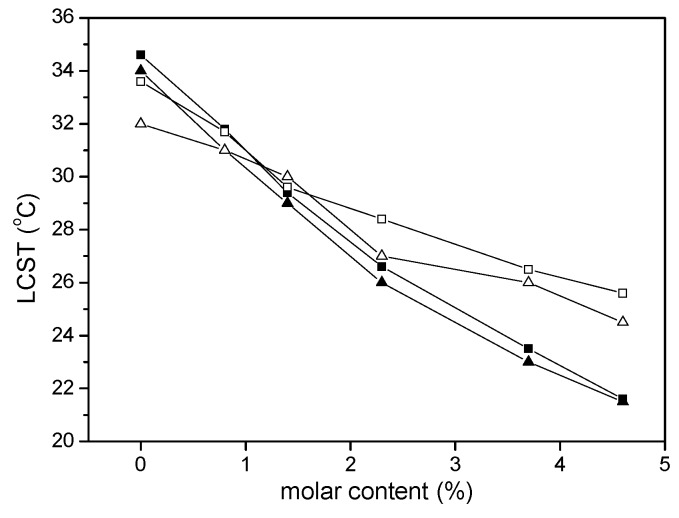
T_cp_ (■ without β-CD, □ with β-CD) and T_ass_ (▲ without β-CD, △ with β-CD) of 5 g/L poly(NIPAM-co-AdMA) aqueous solution.

The temperature and recognition dual responsivity of the poly(NIPAM-co-AdMA) and poly(DMAM-co-AdMA) copolymers was investigated through temperature-dependent transmittance and hydrodynamic diameter (*D*_h_) determined by UV–vis spectroscopy and dynamic light scattering (DLS), respectively. Figure 4 shows a variety of transmittance for 5 g/L poly(NIPAM-co-AdMA) aqueous solution without β-CD (Figure 4a) and with β-CD (Figure 4b) as a function of temperature with a heating rate of 0.5 °C/min. A rapid decline of transmittance could be observed with elevated temperature, indicating that poly(NIPAM-co-AdMA) copolymers were all LCST-type thermoresponsive whether or not β-CD was at present. Furthermore, the *D*_h_ of the aggregates formed by poly(NIPAM-co-AdMA) as a function of temperature at equivalent β-CD or not is shown in Figure 5. The results show that the *D*_h_ of the aggregates had hardly changed in size below the phase transition temperature, and large aggregates appeared and rapidly grew when the temperature reached to the point of phase transition. With further elevated temperature, the *D*_h_ of the aggregates reached maximum and began to decrease, which resulted from further dehydration of the NIPAM unit with increasing temperature [37,38]. T_cp_ and T_ass_ of poly(NIPAM-co-AdMA) copolymers obtained from transmittance and *D*_h_ are depicted in Figure 6. T_cp_ obtained from transmittance was approximately consistent with T_ass_ from the *D*_h_. T_cp_ decreased with increasing content of the AdMA unit in the copolymers, which was attributed to an increase in polymer hydrophobicity by the introduction of the hydrophobic AdMA unit. For the PNIPAM homopolymer, the LCST decreased slightly after the addition of β-CD, probably owing to the enhancing of inter- or intra-polymer hydrophobic interactions, followed by the destruction of partial hydrogen bonding between water and polymers under the action of β-CD. However, for poly(NIPAM-co-AdMA) copolymers, T_cp_ increased with the addition of β-CD and the increment of T_cp_ gradually became large with increasing content of AdMA unit in the copolymers. It is well known that β-CD is water-soluble and capable of selectively including a wide range of hydrophobic guest molecules [39,40]. Adamantyl groups could be included strongly into the cavity of β-CD to form 1:1 complexes [27,28,29,30]. The increase of T_cp_ after the addition of β-CD resulted from the including effect of the hydrophobic adamantyl groups with outer-hydrophilic β-CD. 

**Figure 7:**
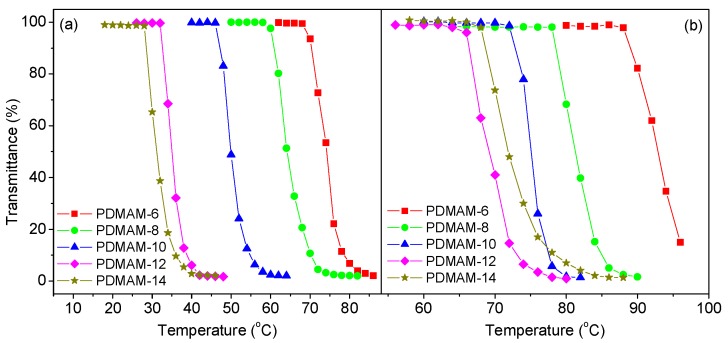
Transmittance as a function of temperature for 5 g/L poly(DMAM-co-AdMA) aqueous solution without β-CD (**a**) and with β-CD (**b**) observed at a wavelength of 600 nm and a heating rate of 0.5 °C/min.

**Figure 8:**
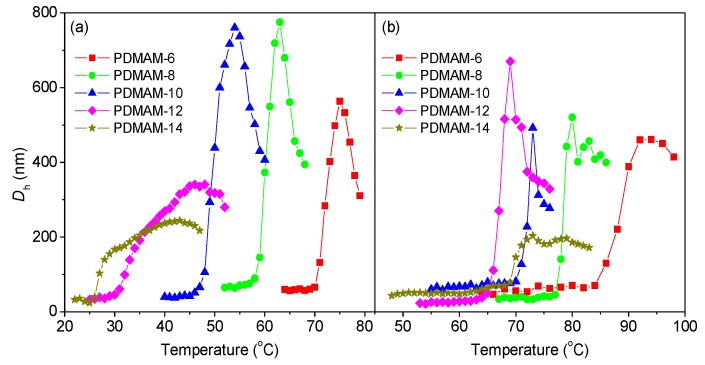
Size distribution of the aggregates of 5 g/L poly(DMAM-co-AdMA) in water without β-CD (**a**) and with β-CD (**b**) as a function of temperature.

**Figure 9:**
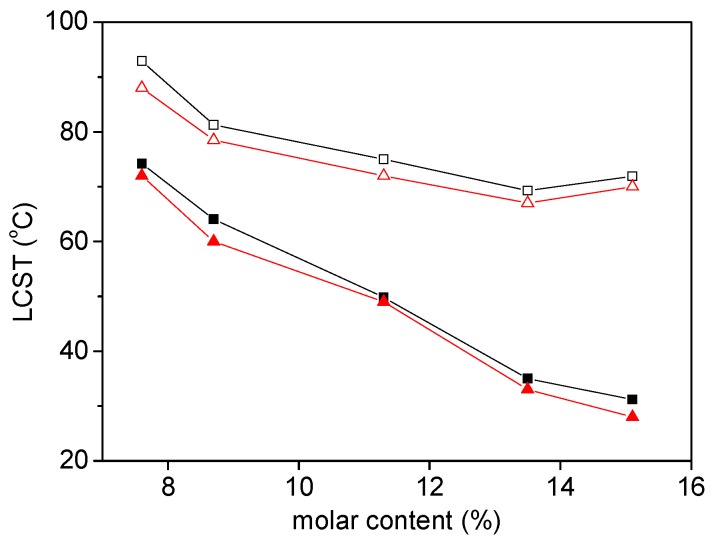
T_cp_ (■ without β-CD, □ with β-CD) and T_ass_ (▲ without β-CD, △ with β-CD) of 5 g/L poly(DMAM-co-AdMA) aqueous solution.

Figure 7 and Figure 8 show the transmittance of poly(DMAM-co-AdMA) aqueous solution and *D*_h_ of the aggregates formed by poly(DMAM-co-AdMA) aqueous solution as a function of temperature in the presence of β-CD or not. Figure 9 depicts the T_cp_ and T_ass_ are related to β-CD and the content of the adamantyl groups in poly(DMAM-co-AdMA). Compared with poly(NIPAM-co-AdMA) copolymers, it needs a higher content of adamantyl groups in the copolymers to endow the poly(DMAM-co-AdMA) copolymers with thermosensitivity. So, poly(DMAM-co-AdMA) copolymers with AdMA unit content from 7.6 to 15.1% were synthesized and used to investigate temperature responsivity. The results of the temperature-dependent transmittance and *D*_h_ show that poly(DMAM-co-AdMA) copolymer aqueous solutions have LCST-type thermosensitivity, the same as poly(NIPAM-co-AdMA). As expected, poly(DMAM-co-AdMA) copolymers exhibited a wide range of phase separation temperature. The T_cp_ of poly(DMAM-co-AdMA) decreased promptly from 74 °C to 31 °C when the content of AdMA unit increased from 7.6 to 15.1% without β-CD. After the addition of β-CD, T_cp_ of poly(DMAM-co-AdMA) increased greatly due to the large polarity transition from hydrophobic adamantyl groups to hydrophilic inclusions of β-CD. It is host–guest recognition of β-CD and adamantyl groups that endows the as-synthesized copolymers with recognition-tunable thermosensitivity. 

## 4. Conclusions

A series of adamantyl-containing poly(NIPAM-co-AdMA) and poly(DMAM-co-AdMA) copolymers with temperature and recognition dual responsivity were successfully prepared by radical copolymerization of NIPAM and DMAM with AdMA monomer. The results show that the as-synthesized copolymers have a lower critical solution temperature (LCST). Phase separation temperature could be determined by either cloud point or DLS. For poly(NIPAM-co-AdMA) and poly(DMAM-co-AdMA), T_cp_ decreases with increasing content of AdMA in the copolymers. After the addition of β-CD, T_cp_ increases, and the increment of T_cp_ gradually became large with increasing content of AdMA in the copolymers. Compared with poly(NIPAM-co-AdMA) copolymers, poly(DMAM-co-AdMA) copolymers exhibit a wide range of phase separation temperature. It is necessary to improve the content of adamantyl groups in the copolymers for achieving recognition-tunable thermosensitivity.

## Figures and Tables

**Table 1:** Polymerization conditions and results of NIPAM, DMAM with AdMA in THF.

Sample	Molar Feed Ratio of AdMA %	Molar Content of AdMA in Copolymer % ^1^	Yield %	Molecular Weight ^2^		
				Mn	Mw	Mw/Mn
PNIPAM	0	0	71	4600	10,800	2.3
PNIPAM-1	1	0.8	58	4000	9700	2.4
PNIPAM-2	2	1.4	62	8600	14,400	1.7
PNIPAM-3	3	2.3	67	5100	10,900	2.1
PNIPAM-4	4	3.7	65	6300	13,400	2.1
PNIPAM-5	5	4.6	71	5900	13,500	2.3
PDMAM	0	0	62	5860	12,300	2.1
PDMAM-6	6	7.6	74	6000	11,500	1.9
PDMAM-8	8	8.7	63	7200	13,700	1.9
PDMAM-10	10	11.3	68	4700	11,700	2.5
PDMAM-12	12	13.5	72	6400	14,800	2.3
PDMAM-14	14	15.1	70	6800	15,100	2.2

^1^ Determined by elemental analysis according to C and N content in the copolymers; ^2^ Determined by GPC based on PS standards in THF.
